# Photodynamic therapy for malignant brain tumors in children and young adolescents

**DOI:** 10.3389/fonc.2022.957267

**Published:** 2022-11-24

**Authors:** Kentaro Chiba, Yasuo Aihara, Yuichi Oda, Atsushi Fukui, Shunsuke Tsuzuki, Taiichi Saito, Masayuki Nitta, Yoshihiro Muragaki, Takakazu Kawamata

**Affiliations:** ^1^ Department of Neurosurgery, Tokyo Women’s Medical University (TWMU), Tokyo, Japan; ^2^ Department of Neurosurgery, Faculty of Advanced Techno-Surgery (FATS), Tokyo Women’s Medical University (TWMU), Tokyo, Japan

**Keywords:** pediatric, adolescent, malignant brain tumor, photodynamic therapy, photosensitizer, laser irradiation

## Abstract

Photodynamic therapy (PDT) targets tumor cell remnants after resection. Here, we evaluated the feasibility of PDT for malignant brain tumors in children and young adolescents. This was a single-center, non-randomized, phase I/II clinical study. The primary endpoints were the safety of treatment with talaporfin sodium (TS) (phase I) and overall survival (OS) after PDT (phase II). The secondary endpoint was progression-free survival (PFS) after PDT. The TS dose was determined by dose escalation from 10 to 20 to 40 mg/m^2^ for every three cases starting from the initial enrolled case. Eight patients with a mean age of 170.2 months (129–214 months) at the time of PDT received nine procedures with a mean follow-up duration of 16.8 months (1–42 months) after PDT. Histopathological diagnoses included supratentorial anaplastic ependymoma (n = 2), anaplastic astrocytoma (n = 1), diffuse midline glioma with H3K27M mutation (n = 1), glioblastoma (n = 3), and pediatric high-grade glioma (n = 1). The outcome was survival in five patients and death in three patients. Recurrence occurred in six of the eight patients; the remaining two were recurrence-free after PDT. Therefore, OS and PFS were calculated as 21 and 6 months, respectively. Seizures and fevers, which were likely surgery-related symptoms, were commonly observed. Photosensitive skin rashes or liver dysfunction, which are common adverse effects in adults, were not observed. Our results showed that TS can be used safely in children at doses comparable to those used in adults, as there was no major complication associated with TS administration. However, we cannot make a definitive conclusion about the efficacy of PDT because of the small number of participants. Accumulating cases was difficult because of the rarity of pediatric brain tumors and the difficulty in making a preoperative differential diagnosis, considering the wide range of histopathological findings. Moreover, the psychological stress associated with light-shielding management in pediatric patients was more severe than initially expected. In conclusion, TS at doses comparable to those used in adults may be safe for use in children and young adolescents between the ages of 6 and 20 years. However, further studies are needed to clarify its efficacy.

## Introduction

Brain and other central nervous system tumors are the most common malignancies in children aged 0–14 years, followed by leukemia (1). In recent years, advances in surgical instruments, assistive devices, and adjuvant therapy have undoubtedly improved the overall survival (OS) and progression-free survival (PFS) for specific types of pediatric brain tumors, although pediatric high-grade gliomas (HGGs) corresponding to WHO grade 3/4 are still refractory to treatment and have a poor prognosis, thus becoming the leading causes of death associated with intracranial disease in children (1–5). For patients in all age ranges, one of the major reasons for the difficulty in treating HGGs is the high incidence of local recurrence (50%–85%) even after maximal surgical resection and subsequent adjuvant therapy, with a PFS rate of 41% at 6 months and 12% at 12 months and an OS rate of 64% at 12 months and 29% at 24 months (2, 4–9). The high local recurrence rate is attributed to the invasive nature of the tumors (2, 7, 9–11). Even if the tumor appears to be completely resected on imaging or microscopy, tumor cells are thought to remain in the normal brain parenchyma surrounding the resection cavity, and recurrence can originate from that site (6, 9–11). Similarly, tumor cells in the eloquent cortex preserved to maintain neurological function and the quality of life (QOL) of children will undoubtedly regrow (6, 8, 9).

Photodynamic (PD) therapy (PDT) is a highly selective treatment method utilizing a photosensitive agent and a specific wavelength laser beam (664 nm) that selectively destroys tumor cells and occludes tumor blood vessels only in tumor tissues, *via* the strong oxidative effect of singlet oxygen generated by photochemical reactions (10–14). At our institution, talaporfin sodium (TS) (Leserphyrin^®^, Meiji Seika Pharma Co., Ltd.) is used as a photosensitive agent (2, 10). In Japan, PDT for lung cancer is already covered by insurance, and it is also indicated for cancers of the trunk, such as esophageal cancer (2, 10, 13, 14). The feasibility of PDT for brain tumors in adults has already been reported by several institutes; however, there are no reports for children (6, 10, 11, 15). The advantages of PDT include good control of local recurrence in the tissue surrounding the resected cavity in cases of gross total resection and the ability to control the regrowth of tumor cells left behind in the eloquent area. Conversely, the disadvantage of PDT, particularly that utilized in the present study, is that the depth of the light is limited to 2.0 to 4.0 mm; therefore, maximum surgical resection is mandatory to achieve optimal effects (6, 10, 13). Recently, the methodological concept of interstitial PDT was introduced, and it is expected that various new methods will be developed to enable the delivery of the PD laser to the target location with equal or better efficacy in the future (16, 17). This will also allow the expansion of the indications of PDT treatment for children and young adolescents, a new age group.

Since the etiological, histopathological, and molecular biological characteristics of pediatric brain tumors differ from those in adults, it has been suggested that the outcomes of PDT in children may be different from those in adults (1, 4, 14, 18–20). The aim of the present study was to evaluate the feasibility, including the safety of TS administration, and efficacy of PDT for pediatric patients.

## Material and methods

This single-center, non-randomized, phase I/II clinical study of PDT for malignant primary brain tumors in children and young adolescents between the ages of 6 and 20 years was approved by the institutional ethics committee (approval no. jRCTs031180360, 4064). Written informed consent was obtained from the parents of all patients who underwent PDT, and written informed assent was obtained from all participants after providing a verbal explanation. Male and female patients who met the following inclusion criteria were eligible for enrollment: treatment on an outpatient or inpatient basis at the Department of Neurosurgery, Tokyo Women’s Medical University Hospital; age of 6–20 years at the time of consent; and suspected primary brain malignancy based on preoperative imaging, with biopsy before resection, or recurrent brain malignancy. Tumors without indications for surgical removal, patients who could not receive TS because of allergies or other reasons, brain tumors other than those with the targeted histopathology, patients who required visual evoked potential monitoring during surgery, pregnant women, patients who were unable to comply with light-shielding management, and patients deemed unsuitable for participation for any reason by the principal investigator were excluded (10, 12, 21).

The TS dose was determined by dose escalation from 10 to 20 to 40 mg/m^2^ for every three cases starting from the initial enrolled case, with a final target of 40 mg/m^2^, which is similar to the dose for adults (10, 12). If an adverse event of grade 3 or higher was observed according to the Common Terminology Criteria for Adverse Events (CTCAE) version 4.0, the dose escalation was sustained (22). If two consecutive patients had grade 3 or higher adverse events, the dose was reduced. Because the assessment of photosensitivity requires at least 2 weeks, new cases were enrolled at least 2 weeks apart. If photosensitivity was observed, the next case was enrolled only after the symptoms disappeared.

**Figure 1:**
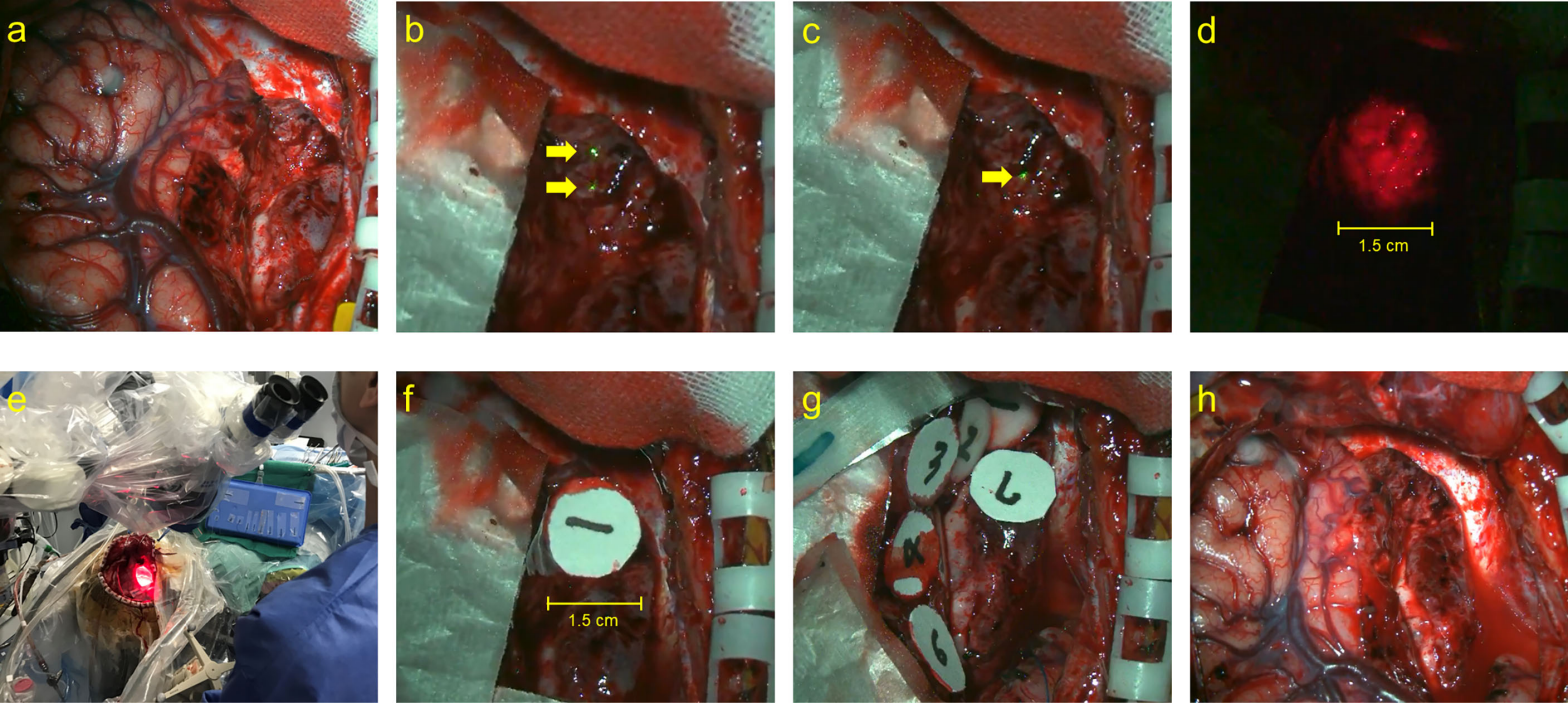
The actual performance of PD laser delivery during PDT. **(A)** The surgical view of tumor resected cavity, **(B)** the two identical green laser beams projected by a laser irradiation unit from a different site (yellow arrows), **(C)** the depth at which the two laser beams intersected in the resection cavity (yellow arrow), **(D)** the irradiation fields of approximately 1.5 cm in diameter, **(E)** the macroscopical surgical view of PDT procedure, **(F, G)** the sterile numbered sheets with the same diameter of 1.5 cm were placed to enable recognition of the irradiated area, and **(H)** the final view of the surgical cavity after PDT.

Similar to that in adults, TS was administered 22–27 h before laser irradiation (10, 12). Light shielding was started immediately after the administration of TS and was continued until day 14 or later when the photosensitivity test was negative on a sunny day. Intraoperative rapid diagnosis was performed for all cases to confirm the intraoperative histopathological diagnosis. Following the maximum possible tumor removal, laser (Panasonic Healthcare Co., Ltd., energy density: 27 J/cm^2^) irradiation was performed (**Figure 1A**) (10, 12). The PD laser irradiation unit was set on the objective lens of the microscope (Mitaka Kohki Co., Ltd., Tokyo, Japan) and connected to a device that provided laser irradiation at a wavelength of 664 nm. An adequate distance between the resection cavity wall and the laser irradiation unit was determined by adjusting the microscope on the basis of two identical green laser beams projected by a laser irradiation unit from a different site; the depth at which the two green laser beams intersected in the resection cavity was considered appropriate (**Figures 1B, C**). When laser irradiation was performed from this distance, it generated irradiation fields of approximately 1.5 cm in diameter on the resection cavity wall; these were confirmed and visualized as red projected areas on the side screen of the microscope (**Figure 1D**). Once the device was turned on, laser irradiation was automatically performed for precisely 3 min; this was considered a single shot (**Figure 1E**). During irradiation, we placed a sterile numbered sheet with the same diameter of 1.5 cm to enable the recognition of the irradiated area (**Figures 1F, G**). These sheets were removed after irradiation was completed (**Figure 1H**). However, the resection cavity was not uniformly flat, and the incidence angle of the laser was occasionally determined by the surgical corridor, considering that some overlap between irradiation fields was unavoidable. The major blood vessels existing within the irradiation field were protected by sandwiched cotton–aluminum foil–cotton sheets until the procedure was completed. During the shading period, the room illumination was set at 500 lx or less, and when it was necessary to transfer patients for examination or other reasons, their whole body was covered with a blanket (12). The site of oxygen saturation monitoring was frequently changed to prevent burns during and after the procedure. After surgery, examination of the pupillary light reflex was avoided as much as possible. Moreover, in conjunction with the evaluation of the side effects of TS administration, the timing of photosensitivity tests was discussed with pharmacologists after checking the weather forecast. After 2 weeks of light-shielding management, the patients were instructed to wear long sleeves, long pants, hats, and sunglasses in the ward until another 2-week indoor shading period was completed (12). Patients could receive adjuvant therapy, including an autologous formalin-fixed tumor vaccine (AFTV), soon after confirmation of the final histopathological diagnosis (10, 23). In case of recurrence, the patient was allowed to receive the treatment of his or her choice without any restriction (10, 11).

The primary endpoints of this study were the safety of TS administration in children and young adolescents (phase I) and OS after PDT (phase II). The secondary endpoint was PFS after PDT.

## Results

**Figure 2:**
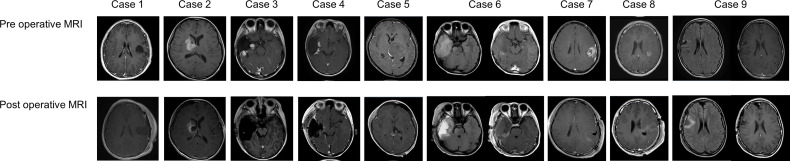
Magnetic resonance imaging findings for all enrolled cases.

A total of nine procedures in eight patients (five boys and three girls) were performed. The mean age at the time of PDT was 170.2 months (range: 129 to 217 months), and the mean follow-up period from TS administration to the last day of follow-up was 18.2 months (range: 1 to 44 months). Five patients were alive at the last follow-up, and three patients succumbed to the primary disease. OS and PFS were 21 and 6 months, respectively. A summary of patient characteristics, neuroimaging modalities, disease states, follow-up periods from initial diagnosis, and PDT and its outcomes is shown in **Figure 2** and **Table 1**. The treatment modalities, including radiation therapy, chemotherapy, AFTV, and stereotactic radiosurgery, as well as the extent of resection, are shown in **Table 2**. A summary of PDT, including the dose assignment, number of PDT shots, photosensitivity test results, and observed symptoms, is shown in **Table 3**. The histopathology and the results of the molecular analysis are summarized in **Table 4**. Biopsy before surgical resection was performed in cases 2 and 8. On average, a photosensitivity test was performed on postoperative day 13.8 (range: days 10 to 16). In many cases, the photosensitivity test was performed on postoperative day 15 because most surgeries were performed on Mondays, and the test could not be performed on Sundays. Furthermore, the timing of the test fluctuated with changes in the weather. In all cases, the photosensitivity test was successfully completed on the first attempt, and none of the patients required a recheck.

**Table 1:** Summary of characteristics of patients, neuroimaging, state of disease, follow-up period, and outcome.

Case	Gender	Age at PDT(mos)	Neuroimaging		Post enhancement on MRIat PDT site	State of disease	PFS (mos)	Follow-up period (mos)		Outcome
			MRS	TNR on methionine PET				From initial diagnosis	From PDT	
1	M	189	Not typical	N/A	Negative	Recurrence	19	53	42	Alive
2	W	135	Not typical	3.01	Negative	Regrowth	44	83	44	Alive
3	W	151	Tumor pattern	N/A	Positive	Dissemination	4	42	21	Dead
4		157	N/A	2.37-4.16	Positive				15	
5	M	129	N/A	5.7	Positive	Residual tumor (two-staged surgery)	8	16	16	Dead
6	M	135	Tumor pattern	2.73	Positive	Recurrence	0	11	1	Dead
7	M	214	N/A	N/A	Positive	Initial	2	28	10	Alive
8	M	205	Tumor pattern	3.78	Negative	Regrowth	4	13	10	Alive
9	W	217	Tumor pattern	3.32	N/A	Recurrence	5	38	5	Alive

M, men; F, women; PDT, photodynamic therapy; N/A, not available; MRS, magnetic resonance spectroscopy; TNR, tumor-to-normal cerebellum ratio; PET, positron-emission tomography; PFS, progression-free survival.

**Table 2:** Treatment modalities and the extent of resection.

Case	Biopsy	EOR	Number of resections	RT	CT	AFTV	SRS
1	(-)	GTR	3	WBI 36.0 Gy/20 fr+focal 18.0 Gy/10 fr	TMZ	(-)	(+)
2	(+)	STR	2	IMRT 54.0 Gy/30 fr	ACNU	(-)	(-)
3	(-)	N/A	4	Brain: IMRT 54.0 Gy/30 fr Spine: 36.0 Gy/20 fr+focal 14.4 Gy/8 fr	BV	(-)	(+)
4							
5	(-)	GTR	3	IMRT 54.0 Gy/30 fr	TMZ/BV	(-)	(-)
6	(-)	GTR	2	IMRT 54.0 Gy/30 fr	TMZ	(-)	(-)
7	(-)	GTR	2	IMRT 60.0 Gy/30 fr	TMZ	(-)	(-)
8	(+)	GTR	4	IMRT 60.0 Gy/30 fr	TMZ/BV	(+)	(-)
9	(-)	GTR	2	IMRT 60.0 Gy/30 fr	ACNU	(+)	(-)

EOR, extent of resection; GTR, gross total resection; STR, sub-total resection; N/A, not available; RT, radiation therapy; WBI, whole-brain irradiation; Gy, gray; fr, fraction; IMRT, intensity-modulated radiation therapy; CT, computed tomography; TMZ, temozolomide; ACNU, nimustine hydrochloride; BV, bevacizumab; AFTV, autologous formalin-fixed tumor vaccine; SRS, stereotactic radiosurgery.

(+), performed; (-), not performed.

**Table 3:** PDT dose assignment, number of shots, photosensitive test results, and symptoms observed after administration of talaporfin sodium.

Case	Dose assignment of talaporfin sodium	Number of PDT shots	Photosensitive test (POD)	Result of photosensitive test	Post enhancement on MRIat PDT site	Symptoms	Characteristics	CTCAE	POD	Allergy	Concomitant medications
1	10	3	13	Negative	Negative	(-)	N/A	N/A	N/A	None	ABPC/SBT, fosPHT, H2 blocker, fentanyl, remifentanil
2	10	1	15	Negative	Negative	Seizure	Left facial, partial seizure	2	6 to 7	Metal	ABPC, fosPHT, H2 blocker, fentanyl, remifentanil, edaravone, acetaminophen, LEV
						Fever	Meningitis	2	20		
3	10	14	16	Negative	Positive	Fever	Meningitis	2	3	Nut	ABPC/SBT, ABK, fosPHT, H2 blocker, fentanyl, remifentanil, acetaminophen, edaravone, CTRX, MEPM
4	20	4	14	Negative	Positive	(-)	N/A	N/A	N/A		ABPC/SBT, ABK, fosPHT, PPI, fentanyl, remifentanil, edaravone, MDZ
5	20	4	15	Negative	Positive	(-)	N/A	N/A	N/A	Edaravone, CTRX, fosPHT	ABPC/SBT, ABK, LEV, H2 blocker, fentanyl, remifentanil, edaravone, metoclopramide
6	20	13	15	Negative	Positive	Palpitation	Spontaneously resolved	1	3	Mackerel	CEZ, LEV, H2 blocker, fentanyl, remifentanil, dexamethasone sodium, edaravone, acetaminophen, MEPM
7	40	6	9	Negative	Positive	Seizure	Short-term generalized convulsive seizure	2	71	None	CEZ, fosPHT, H2 blocker, fentanyl, remifentanil, acetaminophen
8	40	4	15	Negative	Negative	(-)	N/A	N/A	N/A	CTRX, house dust	CEZ, LEV, PPI, fentanyl, remifentanil, edaravone, glycerol, betamethasone, acetazolamide
9	40	4	13	Negative	N/A	Seizure	Left facial, partial seizure	1	6	MRI contrast medium (Magnescope^®^)	CEZ, fosPHT, H2 blocker, fentanyl, remifentanil, metoclopramide, acetaminophen

POD, postoperative day; PDT, photodynamic therapy; N/A, not available; CTCAE, Common Terminology Criteria for Adverse Events, CTRX; ceftriaxone sodium hydrate, fosPHT; fosphenytoin sodium hydrate, ABPC/SBT; ampicillin/sulbactam, LEV; levetiracetam, ABK; arbekacin, MEPM; meropenem, PPI; proton pump inhibitors, MDZ; midazolam.

**Table 4:** Histology and molecular analyses.

Case	Side	Location	Histology	Mib-1LI	Gene panel analysis			
					MS	Mutations	Amplifications	Deletion
1	Left	Frontal lobe	ST-A-EPN	27.7	Stable	*NF2* p.Q319fs*3 *TP53* p.R248W *RB1* p.R556* *BRIP1* p.I952V	Not detected	Not detected
2	Right	Basal ganglia	Anaplastic astrocytoma	1.3	N/A			
3	Right	Frontal lobe	ST-A-EPN	5.7	N/A			
4	Right			11.9				
5	Left	Thalamus	DMG, H3K27-mutant	20-30	Stable	*ATRX* c.5272+2T>A *CEBPA* p.C357fs*42 *ERBB2* p.S1050L *H3F3A* p.K28M *KMT2A(MLL1)* p. L3139F *NBN* p.I171V *TGFBR2* p.S46R *TP53* p.V216M	*PDGFRA* *KIT*	Not detected
6	Right	Parietal lobe	GBM	40	N/A			
7	Left	Parietal lobe	GBM	50-60	Stable	*APC* p.S295G *CARD11* p.S694L *GATA* p.S339R *H3F3A* p.K28M *PDGFRA* p.E229K *PRDM1* p.Q634R *RARA* p.D433E *TP53* p.G108fs*15	*CDK6* *PDGFRA* *KIT* *KDR*	*PDGFRA* intron7/22-intron9/22
8	Left	Parietal lobe	GBM, H3 G34-mutant	50	Stable	*ATRX* p.K562fs*13 *CALR* p.S189N *CDH1* p.V132I *CIC* p.S146fs*1 *CIC* p.G388fs*20 *CIC* p.P1336fs*3 *CXCR4* p.S233T *H3F3A* p.G35R *MTOR* p.I313T *NOTCH1* p.Y550fs*80 *NOTCH3* p.A1450T *SGK1* p.P54T *STK11* p.F354L *TP53* p.R342* *TSC2* p.R245C	Not detected	Not detected
9	Right	Frontal lobe	Pediatric-type diffuse HGA, IDH-1 wild, H3 wild	27	N/A			

ST-A-EPN, supratentorial anaplastic ependymoma; DMG, diffuse midline glioma; GBM, glioblastoma; HGA, high-grade astrocytoma; IDH-1, isocitrate dehydrogenase-1; MS, microsatellite; N/A, not available.

*, termination codon.

**Figure 3:**
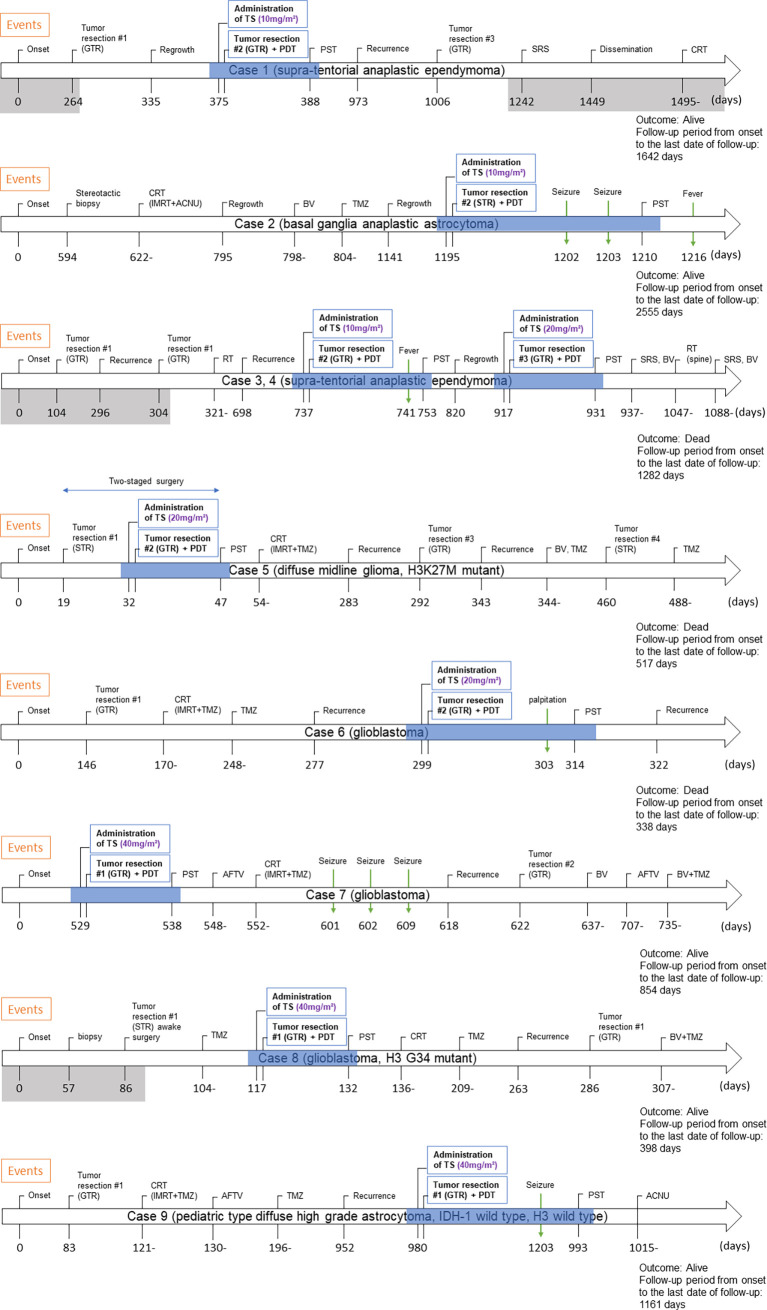
Clinical course of eight pediatric cases who underwent PDT. The period shaded in gray is the period of treatment at the previous hospital. The period during which PDT was performed is shown by blue shading. Abbreviations: GTR, gross total resection; TS, talaporfin sodium; PDT, photodynamic therapy; PST, photosensitivity test; SRS, stereotactic radiosurgery; CRT, chemo-radiotherapy; IMRT, intensity-modulated radiation therapy; ACNU, nimustine hydrochloride; BV, bevacizumab; TMZ, temozolomide; STR, subtotal resection; RT, radiation therapy; AFTV, autologous formalin-fixed tumor vaccine.

Symptoms observed during PDT included convulsions (n = 3), fever (n = 2), and palpitations (n = 1) (**Table 3**). No patient experienced adverse events such as photosensitive skin rashes, liver dysfunction, and shortness of breath, which are well-known complications in adults (10, 11). The clinical course for each case is shown in **Figure 3**.

## Discussion

We conducted a phase I/II clinical study of PDT for malignant brain tumors in children and young adolescents between the ages of 6 and 20 years. Our institution has been providing PDT for adults and has sufficient knowledge regarding its safety and feasibility, as well as the required facilities to manage patients. However, this does not apply to PDT for children. The administration of TS for brain tumors in children has not been reported in the past; therefore, we performed dose escalation involving sequential increases from 10 to 20 to 40 mg/m^2^ for every three patients in the present study (phase I) (21). The major symptoms observed during PDT were partial seizures in three patients and fever in two patients; these were more likely due to the surgical procedure, given the characteristics and timing of onset. Therefore, TS-related adverse events of grade 3 or higher according to CTCAE version 4.0 were not observed (22). These results indicate that TS can be safely utilized in children at a dose of 40 mg/m^2^, which is equivalent to the adult dose (10, 12).

We also tried to clarify the efficacy of PDT for malignant brain tumors in children; however, because of the small number of patients and the heterogeneity in pathological backgrounds, clinical conditions (primary tumor, recurrence, and dissemination), and treatment modalities, including AFTV, further evaluation of safety will be necessary (phase II).

The primary reason for the difficulty in accumulating patients is the rarity of brain tumors in children; the estimated incidence of pediatric brain tumors is 5.85 patients per 10 million children (1). Additionally, pediatric brain tumors have a wider range of histopathological diagnoses, such as low-grade glioma, degenerative diseases representative of multiple sclerosis, demyelinating diseases, and vascular disorders; these occasionally require differentiation from malignant brain tumors (1, 15). Therefore, preoperative differential diagnosis using imaging is very important, but in reality, accurate differentiation of malignant brain tumors indicated for PDT from other brain tumors is difficult. The fact that only one patient underwent PDT at the initial surgical intervention indicates this difficulty; the rest of the cases were those involving recurrence or those involving preoperative biopsy. In addition, the number of patients eligible for PDT is undoubtedly limited because of the tumor location in pediatric patients, who generally develop tumors in the posterior fossa or deeper sites (1, 5, 15, 20). Of course, it is possible to avoid laser irradiation depending on the rapid pathological diagnosis obtained during surgery. Even if TS is administrated, a 1- to 2-week light-shielding period is still necessary; thus, TS administration should be carefully considered after evaluation of preoperative neuroimaging findings (**Figure 2**). Magnetic resonance spectroscopy, [^18^F]fluorodeoxyglucose positron-emission tomography (PET), and 11c-methionine PET are useful neuroimaging modalities for differentiating various diseases; however, their accuracy is inadequate. Thus, we need to accumulate more cases and examine these retrospectively or seek new methods or modalities for the diagnosis of malignant brain tumors with more specificity (7, 9).

In addition to the difficulty in preoperative differential diagnosis, the psychological stress on patients and their guardians due to the light-shielding period and prohibition of the use of electronic devices seemed to be stronger than initially expected, although no case required intervention by a psychologist. The room lighting was dim, under 500 lx, and the use of strong light-producing electronic devices such as phones, computers, and game consoles was prohibited during the shading period. Although the majority of cases completed the 2-week light-shielding period without the photosensitive skin rashes observed in adults, the most common complaint was the inability to use electronic devices, considering that school-aged children in Japan have become dependent on or even addicted to electronic devices of late. Currently, at our hospital, we allow adult patients to use electronic devices after wearing sunglasses and lowering the intensity of the monitor light 1 week after TS administration. The light-shielding protocol may need similar modifications for children in the future.

**Figure 4:**
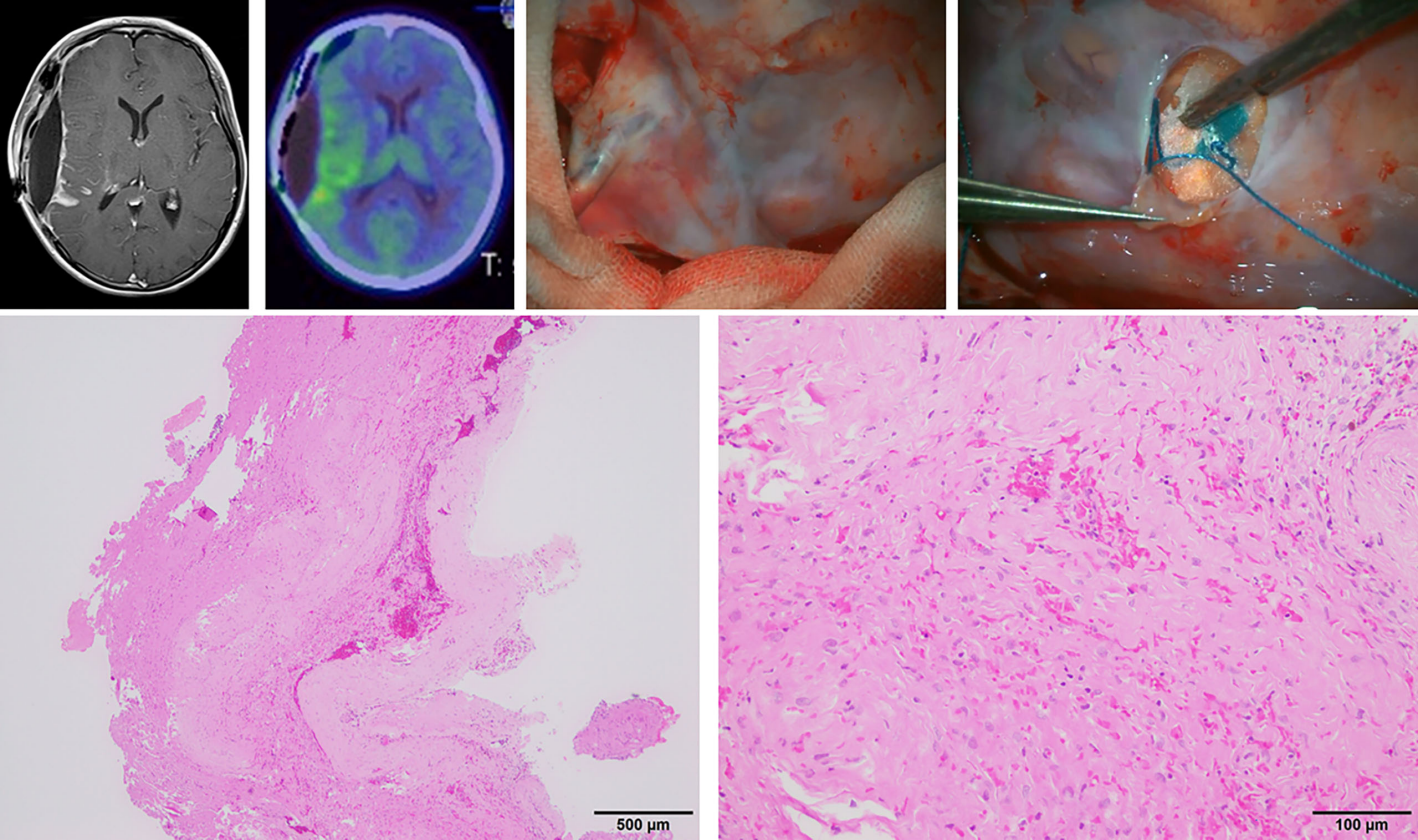
Microscopic view and histopathological findings of a thick membranous tissue on the brain surface, which showed homogenous enhancement on gadolinium-enhanced magnetic resonance imaging on postoperative day 9 in case 3.

Meanwhile, we newly observed a membrane-like area showing homogenous enhancement along the irradiated surface on contrast-enhanced imaging at 1–2 weeks after PDT; this showed spontaneous resolution. A representative case is shown in **Figure 4**, which shows this area on magnetic resonance imaging (MRI) performed on postoperative day 9. In this case, the patient underwent a craniotomy at the time of subsequent recurrence, and a thick coating membrane was observed on the brain surface showing contrast enhancement. Histopathological analysis of this membrane revealed a reactive tissue caused by inflammation, with no obvious infiltration of tumor cells. Although we cannot arrive at a conclusion because of the limited number of affected cases, we suggest that this finding may be related to inflammation caused by the photosensitive reaction (14). Changes in MRI findings after PDT have been reported previously. Aumiller et al. noted the changes in hyperintensity on T1-weighted images after PDT irradiation, which suggested the possibility of PDT-induced deoxygenation of hemoglobin and methemoglobin formation (16). In the future, it will be necessary to clarify whether this feature represents recurrence/dissemination or a benign inflammatory change and identify ways to differentiate these pathologies from each other (14).

There are several unresolved issues in our experience with PDT in children. First, it is necessary to evaluate the extent of TS accumulated within a tissue resected from the surgical site in some manner. TS binds to serum albumin immediately after intravenous injection and is distributed to the tumor cells through the disrupted blood–brain barrier. Then, it is taken up by the tumor cells *via* a specific mechanism (24). It has been reported that TS uptake rates are not uniform among histopathological diagnoses and WHO grades or between primary and recurrent cases (24). Moreover, age could be a factor affecting TS uptake rates. For instance, TS is a water-soluble agent that is primarily distributed in intercellular fluid; therefore, the concentrations may be lower in infants and children, who have a higher percentage of water by weight than adults (24). Furthermore, the main excretion route of TS is biliary excretion. It is generally regarded that renal function is low in neonates and reaches adult levels by 2 years of age, but there are insufficient data on biliary excretory function in children. Thus, it may be necessary to evaluate the rate of TS uptake by tumor cells in pediatric cases in the future. There is a previous report on the utilization of photodynamic diagnosis to prove the accumulation of TS (2, 6, 25). We need to evaluate the concentration of TS accumulated within a resected tissue in order to estimate the optimal timing of TS administration or the appropriate dose more accurately, although we are currently unsure whether the required dose is higher or lower in pediatric patients than in adults. Considering the above, it is possible that a higher dose of TS per body surface area may be required in children than in adults and that the timing of TS administration may need adjustment depending on the results obtained in the future. The second unresolved issue is the ability of methods or devices to deliver the laser light to deeper sites (7). Brain tumors in children occur more frequently at the midline locus, including the thalamus (case 5), basal ganglia (case 2), and brain stem (1, 5, 15). With our method of external irradiation from a PD laser unit installed into a microscope, it is occasionally difficult to deliver the laser beam, which runs straight and forward, through a narrow surgical corridor. The use of mirrors is one possible option, but energy attenuation and blinding may become problematic. It is expected that in the future, laser irradiation using a neuroendoscope, like a bronchoscope, will be able to deliver the laser beam to the resection cavity through a narrow surgical corridor. Currently, PDT treatment for lung cancer has become widely available and is covered by insurance in Japan, and a bronchoscope is utilized for irradiation in clinical practice (10, 14). This will allow visual confirmation of the position and laser irradiation with pinpoint accuracy, even in areas where the brain parenchyma covers and obscures the resection cavity. In addition, as described previously, a new methodological concept of interstitial PDT was recently introduced, and the development of methods or devices for more effective PD irradiation is expected to continue in the future (16, 17). The third unresolved issue is that PDT irradiation takes additional time; one shot of PDT takes 3 min. In case 3, for instance, the patient received 14 shots; thus, the surgery was prolonged by 42 min. Although this additional treatment time will facilitate better tumor control, the treatment goals and priorities should be decided, and random shots should be avoided, with a smooth transition to the next shot to achieve more efficient and effective treatment.

Expansion of indications for younger patients under 5 years and broader histopathological tumor types and tumor locations are primary future expectations (6, 7, 10, 14, 15, 26). Among the various histopathological types of pediatric brain tumors, posterior fossa ependymoma is expected to benefit the most from PDT. There is no standard and effective chemotherapy for ependymoma, and repeated surgical removal is required in cases of recurrence; this is why this disease is referred to as a surgical disease (20, 27–29). Ependymoma has a very high recurrence rate, and aggressive removal is necessary to prevent recurrence; however, the tumor cells are often tightly intertwined with the cranial nerves that control swallowing and speech. Consequently, aggressive removal can cause functional impairment and significantly deteriorate the QOL of the affected child (20, 27, 28). We also experienced a case of postoperative adrenaline-refractory cardiomyopathy due to repeated cardiac arrest and marked blood pressure fluctuations during the detachment of firmly adherent tumor cells in the dorsal medulla oblongata. We expect PDT to affect such residual tumor cells that have been surgically reduced in size at the cranial nerves and medulla oblongata, resulting in better control of tumor progression with minimum neurological deficits. Lowering the age limit for PDT is another problem that needs to be solved as soon as possible because we occasionally encounter brain tumors in infants that are refractory to existing treatment modalities and progress rapidly.

Finally, it is very important to clarify the genetic background of pediatric brain tumors treated with PDT. It is well known that the genetic background of pediatric brain tumors is different from that of adult brain tumors, particularly in terms of the presence or absence of isocitrate dehydrogenase mutations (3, 4, 18, 19, 30). In recent years, gene panel analysis has become widely available. In the present series, gene panel testing was performed for many patients. Different genetic backgrounds may also result in different responses to PDT (14).

## Conclusion

We conducted a phase I/II clinical study of PDT for malignant brain tumors in children and adolescents. No adverse events corresponding to CTCAE grade 3 or higher were observed after dose escalation up to 40 mg/m^2^, which is equivalent to the dose in adults; this suggests that TS can be safely used in children. However, the efficacy of PDT, another objective of this study, could not be demonstrated because of the small number of cases and the diverse clinical backgrounds. Although no major adverse events occurred, the psychological stress during the light-shielding period was greater than initially expected. In the future, it is expected that PDT will be more widely used and will be established as a new add-on treatment that enables the maximum preservation of neurological functions and good local control.

## Data availability statement

The datasets presented in this study can be found in online repositories. The names of the repository/repositories and accession number(s) can be found in the article/supplementary material.

## Ethics statement

The studies involving human participants were reviewed and approved by the Japan Registry of Clinical Trials. Written informed consent to participate in this study was provided by the participant’s legal guardian/next of kin.

## Author contributions

KC wrote the manuscript. YA and YM conceived and designed the clinical study and critically revised the article. AF supported the statistical analysis. TK supervised the study. All the authors contributed to the acquisition of data and read and approved the final version of the manuscript.

## Funding

This study was supported by research funds from the Department of Neurosurgery, Tokyo Women’s Medical University.

## Acknowledgments

We wish to thank Mr. Sakayori (pathological technologist) and the staff of the Department of Neurosurgery and Faculty of Advanced Techno-Surgery (FATS) at Tokyo Women’s Medical University for their guidance and assistance in preparing this paper.

## Conflict of interest

YM reports receiving personal fees from Meiji Pharma Co. The remaining authors declare that the research was conducted in the absence of any commercial or financial relationships that could be construed as a potential conflict of interest.

## Publisher’s note

All claims expressed in this article are solely those of the authors and do not necessarily represent those of their affiliated organizations, or those of the publisher, the editors and the reviewers. Any product that may be evaluated in this article, or claim that may be made by its manufacturer, is not guaranteed or endorsed by the publisher.
