# Corrigendum: Photodynamic therapy for malignant brain tumors in children and young adolescents

**DOI:** 10.3389/fonc.2023.1158407

**Published:** 2023-04-27

**Authors:** Kentaro Chiba, Yasuo Aihara, Yuichi Oda, Atsushi Fukui, Shunsuke Tsuzuki, Taiichi Saito, Masayuki Nitta, Yoshihiro Muragaki, Takakazu Kawamata

**Affiliations:** ^1^ Department of Neurosurgery, Tokyo Women’s Medical University (TWMU), Tokyo, Japan; ^2^ Department of Neurosurgery, Faculty of Advanced Techno-Surgery (FATS), Tokyo Women’s Medical University (TWMU), Tokyo, Japan

**Keywords:** pediatric, adolescent, malignant brain tumor, photodynamic therapy, photosensitizer, laser irradiation

**Incorrect Author Name**

In the published article, an author name was incorrectly written as [Shunsuke Tsuduki]. The correct spelling is [Shunsuke Tsuzuki].

The authors apologize for this error and state that this does not change the scientific conclusions of the article in any way. The original article has been updated.

**Incorrect conflict of interest**

In the published article, there was an error in the “conflict of interest” statement. [The authors declare that the research was conducted in the absence of any commercial or financial relationships that could be construed as a potential conflict of interest.]. The correct Funding statement appears below.

**Conflict of interest**

YM reports receiving personal fees from Meiji Pharma Co.

The remaining authors declare that the research was conducted in the absence of any commercial or financial relationships that could be construed as a potential conflict of interest.

The authors apologize for this error and state that this does not change the scientific conclusions of the article in any way. The original article has been updated.

